# Assessment of Vacuum-Assisted Closure (VAC) Therapy in Orthopaedic Infections

**DOI:** 10.7759/cureus.63204

**Published:** 2024-06-26

**Authors:** Sushil Mankar, Abhijit Kawalkar, Rahul H Sakhare, Pranav Suradkar

**Affiliations:** 1 Orthopaedics and Traumatology, N.K.P. Salve Institute of Medical Sciences & Research Centre (RC) & Lata Mangeshkar Hospital (LMH), Nagpur, IND; 2 Trauma and Orthopaedics, Princess Royal University Hospital, London, GBR

**Keywords:** chronic osteomyelitis, vacuum assisted closure (vac), open fracture wounds, vacuum assisted dressing, negative-pressure wound therapy

## Abstract

Introduction

Chronic and infected orthopaedic wounds may result in profound morbidity, amputation, sepsis and even death. It may need prolonged hospitalization and multiple surgical procedures for treatment. Vacuum-assisted dressing (VAD) is a comparatively newer modality for treating chronic non-healing wounds which helps in faster wound healing, decreases the frequency of dressing and reduces hospitalization time. The aim of our study is to evaluate the outcome of vacuum-assisted dressing (VAD) in the management of orthopaedic wounds.

Materials and methods

A case series including 20 patients with post-traumatic open fracture wounds, post-operative infected wounds and wounds with underlying chronic osteomyelitis were treated with VAD. Wound size was measured pre- and post-debridement and every five days, at the time of dressing change until the wound healed or grafted. The duration of wound healing or wound closure was measured and documented.

Results

Wound size decreased significantly and healthy granulation tissues were observed in all wounds after the application of vacuum-assisted dressing. Wound size decreased by an average of 22% after debridement and first vacuum-assisted dressing removal. Infection control was achieved in 18 out of 20 patients (90%) who had wounds closed either by secondary closure or by skin grafting.

Conclusion

We conclude that VAD is an efficient technique in the management of orthopaedic wounds, especially in the management of open fracture wounds but less effective in chronically infected wounds with underlying osteomyelitis.

## Introduction

Chronically infected wounds after open fractures and post-orthopaedic surgical procedures are worrisome complications encountered by orthopaedic surgeons. These wounds are the major cause of morbidity and also reason for prolonged hospitalization thus increasing the cost of treatment among orthopaedic patients [1]. Wound healing is a complex process which includes removal of dead tissues, control of infection, increased blood flow and formation of granulation tissue [2]. Infections complicate the treatment of wounds by impeding the healing process. When bacteria begin replication in the wound and increase their metabolic activity, the resulting toxins negatively impact all phases of wound healing [3].

Vacuum-assisted dressing (VAD) also referred to as vacuum-assisted closure (VAC) or negative pressure wound therapy (NPWT), or micro deformational wound therapy (MDWT) is a comparatively newer modality for treating chronic non-healing wounds. First described by Fleischmann et al. [1] in 1993, it employs the application of constant sub-atmospheric pressure on the wound which leads to reduction in wound size, stimulation of granulation tissue, removal of small tissue debris, decreased protease content and removal of exudate. It also reduces interstitial oedema thereby increasing microcirculation, local blood flow and oxygenation [4].

Negative pressure wound therapy (NPWT) is a well-known modality in the management of open fracture wound but it has not been very well studied in chronically infected orthopaedic wounds. This study aims to evaluate the outcome of vacuum-assisted dressing (VAD) in various aspects of orthopaedic practice from chronic wounds with underlying osteomyelitis to open fracture wounds.

## Materials and methods

A case series including 20 patients was carried out between April 2019 and October 2021 in a tertiary care hospital. We included post-traumatic open fracture wounds, post-operative infected wounds and wounds with underlying chronic osteomyelitis. We excluded patients with superficial skin wounds and wounds with exposed nerves or large vessels.

Institutional ethics committee approval was obtained and a total of 20 patients were included in the study. Informed written consent was taken from all patients. We had nine patients with open fracture wounds, six patients had post-op infected wounds and five patients had chronic wounds with underlying osteomyelitis. All wounds were thoroughly debrided before the application of vacuum-assisted dressing and tissue samples were sent for microscopy, cultures and sensitivity testing. Subsequent wound debridement and vacuum-assisted dressings were applied as needed. Wound size was measured pre- and post-debridement and every five days, at the time of dressing change until the wound healed or grafted. Wound cultures were taken at the time of first debridement and when the last vacuum-assisted dressing was removed. The open fracture group received antibiotics prior to the VAC therapy but for patients with post-op wound infections, the antibiotics were started after the wound swab was taken. In cases of chronic wounds patients did not have antibiotics for at least one week before the wound swabs, but were restarted after the VAC application (Table 1).

**Table 1:** Pre- and post-VAD treatment organisms isolated from the wound cultures. VAD: Vacuum-assisted dressing

Culture organism	Pre-VAC	Post-VAC
No growth	11	18
Staphylococcus aureus (gram-positive)	2	0
Streptococcus pyogenes (gram-positive)	3	0
Pseudomonas aeruginosa (gram-negative)	1	1
Klebsiella pneumoniae (gram-negative)	2	1
Non-fermenter species (Acinetobacter- gram-negative)	1	0

The duration of wound healing or wound closure was measured and documented.

The vacuum-assisted dressings were changed every five days and the wound was examined for its dimensions, appearance and signs of infection. The endpoint of negative pressure wound therapy was when there were no signs of infection and the wound could be closed by secondary suturing or when the wound was ready for grafting. All patients received broad-spectrum antibiotics (ceftriaxone+ sulbactam and clindamycin) before the culture reports which were changed to sensitive antibiotics once the reports were available. Broad-spectrum antibiotics were continued for the patients in whom no bacteria were grown on cultures in case of chronic wounds. In patients with open fractures, antibiotics were continued for three days post-debridement and VAD application.

## Results

Our study included 20 patients - 13 males and seven females - with a mean age of 39 years. The average period of the application of vacuum-assisted wound dressing was 12.5 days. All open fracture wounds (7 grade IIIA and 2 grade IIIB) healed in one to two (5-10 days) VAD applications or were ready for grafting. Four out of six post-operatively infected wounds healed with two VADs but the remaining two needed three VADs, but all six wounds healed and infection was controlled. Chronic wounds with underlying osteomyelitis needed at least three VADs and two out of five wounds did not heal even after four settings and negative pressure wound therapy was abandoned for these two patients who remained chronically infected even after repeated debridement and four VADs (Table 2).

**Table 2:** Etiology, mean age and outcomes of VAD. VAD: Vacuum-assisted dressing

Etiology	
Open fracture wounds	9
Post-operative infected wounds	6
Chronic infection with osteomyelitis	5
Mean age (In years)	39 (Range: 18-68)
Mean decrease in wound size after 1^st^ VAD	22% (Range: 15-34%)
Mean duration of VAD application (In days)	12.5 (Range: 5-20)
Method of wound closure	
Secondary suturing	12
Split-thickness skin graft	4
Myo-cutaneous flap (by plastic surgeon)	2

**Figure 1:**
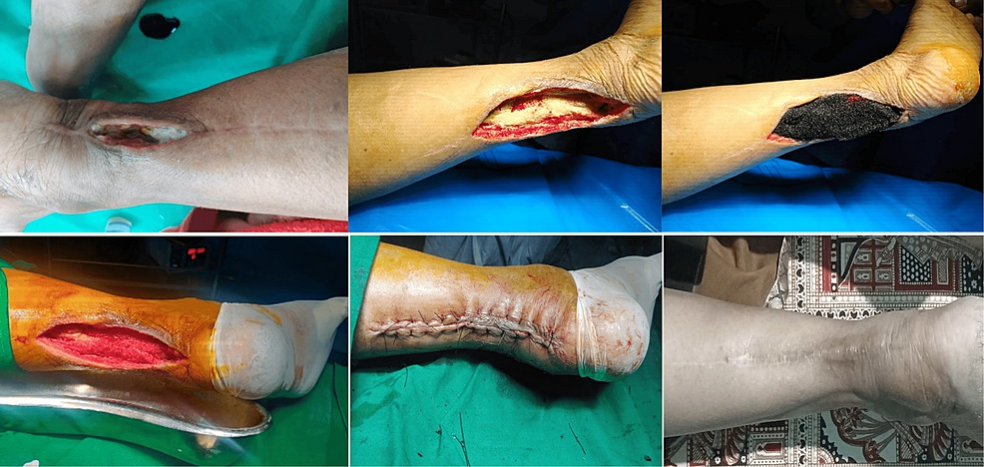
Case 1: NPWT in infected wound of a Tendo Achilles repair procedure (secondary closure at the end of 2 VADs) NPWT: Negative pressure wound therapy; VAD: Vacuum-assisted dressing

**Figure 2:**
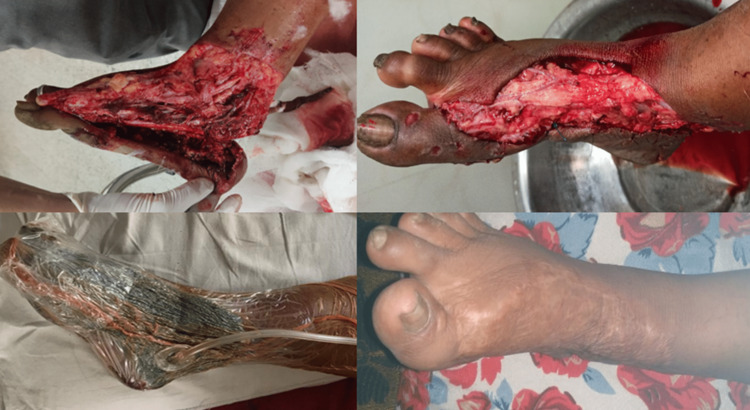
Case 2: NPWT in open grade III B degloving wound of the foot with heel pad avulsion (Skin graft for closure at the end of 2 VADs) VAD: Vacuum-assisted dressing; NPWT: Negative pressure wound therapy.

In our study, it is observed that wound size decreased significantly, and healthy granulation tissues were observed in all wounds after the application of vacuum-assisted dressing. Wound size decreased by an average of 22% (range 15%-34%) after debridement and first vacuum-assisted dressing removal. Infection control was achieved in 18 (90%) of our patients who had wounds closed either by secondary closure, split-thickness skin grafting or by myo-cutaneous graft (with the help of plastic surgeons) (Figures 1, 2). We consider this as a successful outcome. Two patients with chronic wounds with underlying osteomyelitis had persistent infection with chronically discharging wounds/sinuses, but even in these cases the wound size decreased and healthy granulation tissue was formed. We closed both these wounds with secondary suturing but both wounds gapped and cultures grew the same organisms as before. We considered this as a failure of vacuum-assisted dressing treatment.

## Discussion

Negative pressure wound therapy has been extensively studied in the management of open fracture wounds in orthopaedics. It reduces the risk of infection and accelerates the wound-healing process [5]. In open wounds, NPWT promotes wound healing by facilitating the removal of excess interstitial fluid, reducing oedema, promoting fluid drainage, improving lymphatic flow, decreasing hematoma and seroma formation and enhancing tissue growth, and expansion [6,7]. This study provides evidence that VAD can be extended for the management of post-operative wound infections in orthopaedics and chronic wounds with underlying osteomyelitis along with the management of open fracture wounds.

Open fractures with large defects have high chances for delay and non-union, also open wounds are prone to infection and should be protected from environmental bacteria while promoting drainage [8]. Early coverage of bones and tendons is crucial for achieving good outcomes. NPWT therapy helps reduce the time between initial debridement and the definitive procedure to cover the wound [9]. NPWT/VAC compared to the traditional wound dressings showed better results in terms of the appearance of granulation tissue, reduction in wound surface area and duration of hospital stay [10]. NPWT for open wounds at the fracture site has been shown to prevent infection [11, 12]. NPWT may increase blood flow in the vicinity of the open wound and contribute to granulation tissue formation and prevention of surgical site infection.

In our study, all nine patients with open fracture wounds had successful outcomes with control of infection and definitive wound closure with two settings of VAD. However, NPWT was successful only in three out of five patients with chronic wounds with underlying osteomyelitis bringing down the success rate to only 60%, which indicates that it can be used with very high success in selected groups of wounds. We also sent wound swabs and/or tissues for culture and sensitivity at the time of first debridement and after the last VAD was removed to assess the eradication of infection from the wound after NPWT but as 11 out of 20 patients had negative cultures at the time of first debridement, we did not have sufficient data for statistical analysis.

The most important advantage of VAD is that it needs less frequent dressing changes as compared to conventional wound dressings [13], we changed VAD every five days, and these dressing changes can be done in the ward if patients do not need debridement of the wound. This is particularly important as it reduces the workload for the health care staff and in the wounds which are difficult to dress. The potential downside of the NPWT is rapid loss of volume and electrolytes in very oozy wounds and post-VAD blood loss in patients with coagulopathy. In both situations, patients' VAD drain output needs to be closely monitored along with their haemoglobin and electrolytes [14]. Other potential risks are skin maceration and skin blister formation [15], but we think that careful application of adhesive dressing and properly drying the skin before the placement of adhesive drape helps minimise this risk. We did not have any skin maceration or blisters in our study.

We acknowledge that our study has a relatively small number of cases, however, complex wounds are relatively rare and it is exceedingly difficult to have a significantly higher number of cases in the study. Additionally, as it is not a comparative study, we do not have statistical data that directly compare the clinical results of traditional wound management techniques used in this condition. To obtain more definitive results, a larger multicentric comparative study is needed.

## Conclusions

Our study concludes that NPWT is an efficient technique in the management of orthopaedic wounds, especially in the management of open fracture wounds but it is by no means a panacea for wound management. It can also be used in the initial management of chronically infected wounds with underlying osteomyelitis but has a limited role and low success rate in the control of infection. NPWT helps to reduce the wound size significantly and helps in the early closure of the wounds.
